# Supplementary material for: Study on the Efficacy and Safety of Tedizolid in Japanese Patients
Source: Antibiotics (Basel). 2024 Dec 23;13(12):1237. doi: 10.3390/antibiotics13121237 (PMC11672433; doi:10.3390/antibiotics13121237)
Supplement: Supplementary file 1 [file antibiotics-13-01237-s001.zip › antibiotics-3312785-supplementary.pdf]

Supplemental Table S1

| Underlying diseases of liver dysfunction | n=9 episodes |
|---|---|
| NASH, Fatty liver, n(%) | 2(22.2) |
| HBV, n(%) | 1(11.1) |
| other, n(%) | 6(66.7%) |
| **Underlying diseases of heart failure** | **n=19 episodes** |
| CHF, n(%) | 4(21.1) |
| HTN, n(%) | 1(5.3) |
| multiple factors, n(%) | 3((15.8) |
| unknown, n(%) | 11(57.9) |
| **hematological malignancy** | **n=13 episodes** |
| AML, n(%) | 7(53.8) |
| ALL, n(%) | 3(23.1) |
| MDS, n(%) | 1(7.7) |
| AITL, n(%) | 2(15.4) |
| HSCT, n(%) | 4(30.8) |

Supplemental Table S2

| | n=40 episodes |
|---|---|

| | |
|---|---|
| WBC, median (/μL) (IQR),<br>WBC ≤500/μL, n(%) | 5.25(4.98)<br>4(10) |
| Hgb, median (g/dL) (IQR)<br>Hgb ≤7.0 g/dL, n (%) | 9.75(3.75), 4(10) |
| PLT, median (/μL) (IQR)<br>PLT ≤50,000/μL, n (%) | 12.9(19.4), 10(25) |
| Serum Cr, median (mg/dL) (IQR) | 1.15(1.10) |
| AST, median (U/L) (IQR) | 27.0(23.8) |
| ALT, median (U/L) (IQR) | 18.5(22.3) |
| G-GTP, median (U/L) (IQR) | 42.0(60.0) |
| T-bil, median (mg/dL) (IQR) | 0.600(0.60) |
| ALP (IFCC), median (U/L) (IQR) | 93.5(71.3) |
| LAC, median (mg/dL) (IQR) | 1.40(1.33) |
| qSOFA score ≥2, n (%) | 7(17.5) |

Supplemental Table S3

| | n=40 episodes |
|---|---|
| WBC, median (/μL) (IQR)<br>WBC ≤500/μL, n (%) | 5.55(4.90)<br>1(2.5) |
| Hgb, median (g/dL) (IQR)<br>Hgb ≤7.0 g/dL, n (%) | 9.40(4.85)<br>7(17.5) |
| PLT, median (/μL) (IQR)<br>PLT ≤50,000/μL, n (%) | 14.2(17.1)<br>9(22.5) |

| | |
|---|---|
| Serum Cr, median (mg/dL) (IQR) | 1.00(0.96) |
| AST, median (U/L) (IQR) | 26.0(25.8) |
| ALT, median (U/L) (IQR) | 22.0(22.3) |
| G-GTP, median (U/L) (IQR) | 40.0(113) |
| T-bil, median (mg/dL) (IQR) | 0.600(0.725) |
| ALP (IFCC), median (U/L) (IQR) | 99.9(78.1) |
| LAC, median (mg/dL) (IQR) | 1.40(1.30) |

Supplemental Table S4

| | n=40 episodes | Missing values |
|---|---|---|
| WBC, median (/μL) (IQR) | 5.80(5.65) | 19 |
| Hgb, median (g/dL) (IQR) | 11.3(4.45) | 23 |
| PLT, median (/μL) (IQR) | 17.4(18.4) | 23 |
| Serum Cr, median (mg/dL) (IQR) | 0.98(1.1) | 23 |
| AST, median (U/L) (IQR) | 18.0(21.5) | 23 |
| ALT, median (U/L) (IQR) | 18.0(13.5) | 23 |
| G-GTP, median (U/L) (IQR) | 28.0(19.0) | 25 |
| T-bil, median (mg/dL) (IQR) | 0.600(0.500) | 25 |

| | | |
|---|---|---|
| ALP (IFCC), median (U/L) (IQR) | 79.8(27.5) | 24 |
| LAC, median (mg/dL) (IQR) | 1.00 | 37 |

Supplemental Table S5

| Case | hematological malignancy | PLT on admission | PLT at the end of treatment | ICU admission/Vasopressor use/Mechanical ventilator/mortality | PLT transfusion/G-CSF |
|---|---|---|---|---|---|
| 81 y, female, BMI 24.0, Cr 1.92, tedizolid for 6 days | ALL | 12.9 | 3.2 | yes/yes/yes/dead | yes/yes |
| 64 y, man, BMI 26.4, Cr 6.2, 12 days | AML, HSCT | 6.5 | 3.5 | yes/yes/yes/alive | no/no |
| 69 y man, BMI 18.9, Cr 0.61, 7 days | AITL, HSCT | 5.1 | 3.7 | no/no/no/dead | yes/yes |
| 55 y man, BMI19.7, Cr 1.14, 12 days | AML | 6.3 | 3.4 | yes/no/yes/alive | yes/no |

Supplemental Table S6

| Case laboratory test at the time of administration | cause of discontinuation | duration of treatment | Concomitant medications | hematological malignancy | AST/ ALT at the end of treatment | G-GTP/ALP/ T-bil at the end of treatment | ICU admission / Vasopressor use / Mechanical ventilation / mortality | Transfusion / G-CSF use |
|---|---|---|---|---|---|---|---|---|
| 79 y, man, BMI19.5, AST 21 ALT 28 G-GTP 18 ALP139(IFCC) T-bil 0.4 | hepatic dysfunction | 9 days | TPN, fat emulsion, VCM, Posaconazole | no | 169/221 | 153/423 (IFCC) /0.3 | no/no/no/ alive | no/no |

Supplemental Table S7

| case | Age/ Sex | duration (days) | hematological malignancy | diagnosis of infection | transfusion | prognosis |
|---|---|---|---|---|---|---|
| Case 1 | 80/F | 118 | 0 | MRSA septic arthritis | RCC 4unit/PLT 10unit | alive |
| Case 2 | 76/F | 77 | 0 | Septic arthritis | RCC 16unit | alive |
| Case 3 | 85/F | 28 | 1 | E. faecium nosocomial meningitis | | dead(non-infection) |
| Case 6 | 64/M | 55 | 0 | MRCNS CRBSI | | alive |
| Case 7 | 69/M | 28 | 0 | MRSA prosthetic intravascular infection | | alive |
| Case 13 | 82/M | 42 | 0 | MRSA osteomyelitis | | alive |
| Case 14 | 89/F | 107 | 0 | Th11, 12, L1osteovertebritis | RCC 4unit | alive |
| Case 15 | 72/F | 81 | 0 | MRSA SSI/osteomyelitis | | alive |
| Case 19 | 76/F | 203 | 0 | SSI/osteomyelitis | | alive |
| Case 29 | 17/F | 66 | 0 | MRSASeptic arthritis | | alive |
| Case 30 | 47/M | 51 | 0 | MRSA subcutaneous/intramuscular abscess | | alive |
| Case 31 | 73/F | 69 | 0 | SSI/osteomyelitis | | alive |
| Case 36 | 44/F | 77 | 0 | SSI/Intrauterine infection | | alive |

| | | | | | |
|---|---|---|---|---|---|
| Case 37 | 49/M | 76 | 0 | MRSAseptic arthritis・prosthetic intravascular infection | alive |

Supplemental Table S8

| | non-Hematologic Malignancies: (n=27) | Hematologic Malignancies:(n=13) | p |
|---|---|---|---|
| WBC, median (/µL) (IQR),<br>WBC ≤500/µL, n(%) | 5.00(4.20), 0(0) | 5.50(14.5), 4(30.8) | 0.713 |
| Hgb, median (g/dL) (IQR)<br>Hgb ≤7.0 g/dL, n (%) | **10.7(3.60), 1(3.7)** | **7.90(2.05), 3(23.1)** | **<0.01** |
| PLT, median (/µL) (IQR)<br>PLT ≤50,000/µL, n (%) | **188(133), 2(7.4)** | **42.0(40.5), 8(61.5)** | **<0.01** |
| Serum Cr, median (mg/dL) (IQR) | 0.84(0.83) | 1.74(0.91) | 0.089 |
| AST, median (U/L) (IQR) | 21.0(17.0) | 44.0(69.0) | 0.20 |
| ALT, median (U/L) (IQR) | 19.0(21.0) | 18.0(32.5) | 0.716 |
| G-GTP, median (U/L) (IQR) | **34.5(34.0)** | **109.0(445)** | **0.009** |
| T-bil, median (mg/dL) (IQR) | 0.4(0.4) | 0.9(3.15) | 0.055 |
| ALP (IFCC), median (U/L) (IQR) | **82.6(27.0)** | **221(191)** | **0.023** |
| LAC, median (mg/dL) (IQR) | 1.2(0.950) | 1.85(1.65) | 0.211 |
| qSOFA score ≥2, n (%) | 1(3.7) | 6(46.2) | 0.078 |

Supplemental Table S9

| | non-Hematologic Malignancies: (n=27) | Hematologic Malignancies:(n=13) | p |
|---|---|---|---|

| | | | |
|---|---|---|---|
| WBC, median (/µL) (IQR) | 5.60(2.80) | 3.90(8.50) | 0.769 |
| WBC ≤500/µL, n (%) | **0(0.0)** | **1(7.7)** | |
| Hgb, median (g/dL) (IQR) | **11.5(4.5)** | **7.9(2.15)** | **<0.01** |
| Hgb ≤7.0 g/dL, n (%) | **2(7.4)** | **5(38.5)** | |
| PLT, median (/µL) (IQR) | **188(123)** | **34.0(32.5)** | **<0.01** |
| PLT ≤50,000/µL, n (%) | **0(0.0)** | **9(69.2)** | **0.031** |
| Serum Cr, median (mg/dL) (IQR) | 0.790(0.590) | 1.42(1.55) | 0.185 |
| AST, median (U/L) (IQR) | 22.0(15.0) | 45.0(63.0) | 0.205 |
| ALT, median (U/L) (IQR) | 20.0(20.0) | 24.0(39.5) | 0.875 |
| G-GTP, median (U/L) (IQR) | **39.0(61.5)** | **129.0(254.5)** | **0.045** |
| T-bil, median (mg/dL) (IQR) | **0.40(0.35)** | **1.10(5.25)** | **0.020** |
| ALP (IFCC), median (U/L) (IQR) | **89.3(29.4)** | **182(178)** | **0.039** |
| LAC, median (mg/dL) (IQR) | 1.10(0.800) | 1.60(1.85) | 0.186 |

Supplemental Table S10

| | non-Hematologic Malignancies: (n=27) | Hematologic Malignancies:(n=13) | p |
|---|---|---|---|
| WBC, median (/µL) (IQR) | 6.45(5.10) | 4.2(un-estimated) | 0.772 |
| Hgb, median (g/dL) (IQR) | **12.0(4.40)** | **8.25(un-estimated)** | **<0.01** |

| | | | |
|---|---|---|---|
| PLT, median (/µL) (IQR) | **176(145)** | **43.5(un-estimated)** | **<0.01** |
| Serum Cr, median (mg/dL) (IQR) | 0.77(1.07) | 2.145(un-estimated) | 0.483 |
| AST, median (U/L) (IQR) | 18.0(16.0) | 34.0(un-estimated) | 0.720 |
| ALT, median (U/L) (IQR) | 18.0(19.0) | 17.5(un-estimated) | 0.264 |
| G-GTP, median (U/L) (IQR) | 28.0(31) | 32.0(un-estimated) | 0.294 |
| T-bil, median (mg/dL) (IQR) | 0.60(0.45) | 2.5(un-estimated) | 0.509 |
| ALP (IFCC), median (U/L) (IQR) | 79.8(23.8) | 116(un-estimated) | 0.598 |
| LAC, median (mg/dL) (IQR) | un-estimated | un-estimated | |
